# Editorial: Neutrophil Communication

**DOI:** 10.3389/fimmu.2020.00871

**Published:** 2020-04-30

**Authors:** Jason S. Knight, Rohit Jain, Marco A. Cassatella, Christian Lood

**Affiliations:** ^1^Division of Rheumatology, Department of Internal Medicine, University of Michigan, Ann Arbor, MI, United States; ^2^Faculty of Medicine and Health, Centenary Institute, The University of Sydney, Camperdown, NSW, Australia; ^3^Section of General Pathology, Department of Medicine, University of Verona, Verona, Italy; ^4^Division of Rheumatology, Department of Medicine, University of Washington, Seattle, WA, United States

**Keywords:** neutrophil, malignancy, platelet, autoimmunity, infection, inflammation, cell-cell interaction, neutrophil extracellular traps

Neutrophils are key immune cells that participate in host defense through a variety of mechanisms including phagocytosis, the generation of reactive oxygen species (ROS), release of granular contents, and the formation of neutrophil extracellular traps (NETs). The capacity of neutrophils to orchestrate inflammatory and immune responses is dependent on their release of neutrophil-derived molecules, including cytokines, alarmins, and NETs, as well as their capacity to interact with and direct other innate and adaptive immune cells. Over the past decade, the field of neutrophil biology has exploded, with remarkable discoveries highlighting neutrophils as indispensable players in immune regulation. While neutrophils are recognized to be prominent phagocytes involved in the clearance of pathogens and cell debris, they are beginning to emerge as essential communicators crucial in shaping immune response.

In this Research Topic, a series of articles provides comprehensive insights into the current view of neutrophil biology, highlighting neutrophil interactions with platelets, infectious agents, and tumor cells, as well as neutrophil generation of inflammatory mediators such as NETs.

In the review by Wirestam et al., we are reminded of the devastating role of neutrophils in rheumatic diseases, including systemic lupus erythematosus (SLE) and anti-phospholipid syndrome (APS) wherein neutrophils recognize circulating immune complexes promoting ROS-dependent NET formation. Intriguingly, SLE patients have impaired NOX2, relying on mitochondrial ROS for NET formation. This results in concomitant mitochondrial extrusion and a qualitatively distinct NETs with inflammatory oxidized mitochondrial DNA as an important cargo. Takeda et al. highlighting the important role for immune complexes, namely bactericidal/permeability-increasing protein (BPI) immune complexes, in mediating NET formation in systemic anti-neutrophil cytoplasmic antibody (ANCA)-associated vasculitis. The regulation and role of the main immune complex receptor, Fc gamma R, in steady state and inflammatory conditions, is comprehensively covered in an excellent review by Wang and Jönsson. Beyond immune complexes, large aggregates, including monosodium urate (MSU) crystals are known to activate macrophages and neutrophils, though the underlying signaling events have not been well-characterized. In an elegant study by Tatsiy et al., performing transcriptomic analyses, the authors demonstrate the novel finding that neutrophils, upon engulfment of MSU crystals, are programmed to attract monocytes in an NF-κB- and CCL4-dependent manner. This observation is particularly interesting as neutrophils are essential in licensing macrophages for cytokine production in atherosclerosis, indicating a pathophysiological role for their crosstalk (1). Furthermore, the authors find that TAK1 and Syk are early mediators, upstream of MAPK and Akt, partaking in NET formation, and as such, representing a potential therapeutic target for gouty arthritis. A similar transcriptomic approach was undertaken by Miralda et al. to assess neutrophil signaling pathways regulated by the bacteria *Filifactor alocis*. Among the many pathways being regulated by *F. alocis*, the authors made the intriguing finding that the bacteria blunted TNF-induced MAPK activation in neutrophils, suggesting that this may be a bacterial strategy to subvert innate immunity.

Another study investigating the underlying requirements for NET formation (Gößwein et al.), made the novel observation of a crosstalk between a protease, calpain, and peptidylarginine deiminase 4 (PAD4) in nuclear decondensation, with PAD4-mediated citrullination facilitating proteolytic cleavage of nuclear proteins. This observation markedly advances our understanding of the molecular events during NET formation and may allow for additional therapeutic strategies to prevent exacerbated NETosis. In the final NET-related paper included in this Research Topic, Agarwal et al. demonstrate that NETs are not only a product of neutrophil activation, but also a potent stimulus for secondary NETosis, inducing NET formation through TLR9-dependent mechanisms. This mechanism may be particularly important in post-traumatic inflammation, when NETs disrupted by mechanical strain may amplify inflammation and prevent physiological wound healing.

Two interesting papers in this collection suggest a critical role for platelets as orchestrators of neutrophil recruitment to diseased organs. Psoriasis is a systemic inflammatory disease characterized by intense leukocyte infiltration into the skin. Herster et al. undertook an unbiased screen of neutrophil surface markers in individuals with psoriasis and found upregulation of CD41 and CD61 (two classic platelet glycoproteins). These surface markers were attributable to increased platelet-neutrophil aggregates in the blood of patients with psoriasis (Herster et al.). The aggregates also appeared to infiltrate the skin, where platelets could be found in close proximity to both intact neutrophils and neutrophil remnants suggestive of NETs (Herster et al.). Intriguingly, platelet depletion was protective against neutrophil infiltration and overall skin inflammation in a mouse model of psoriasis (Herster et al.).

Systemic inflammatory response syndrome (SIRS) puts many organ systems at risk including the lungs, where it may lead to acute respiratory distress syndrome. Hook et al. characterized a model of SIRS triggered by administration of zymosan. This model has previously been shown to be exacerbated by NADPH oxidase 2 (Nox2) deficiency. In this new study (Hook et al.), Nox2-deficient mice demonstrated upregulation of platelet-derived chemokines such as CXCL4 and CXCL7 in the bronchoalveolar fluid. Neutrophils strongly expressing P-selectin glycoprotein ligand-1 were found at increased levels in the same fluid (Hook et al.). While Nox2 deficiency may protect against NETosis in some contexts, that was not the case in this model where exaggerated NETosis appeared to be driven by platelets in a PAD4-dependent fashion (Hook et al.). In summary, these studies shine a light on the role of platelets in recruiting neutrophils into inflamed tissues, and suggest the potential for anti-platelet agents to have efficacy in neutrophil-dependent diseases.

A further group of articles have focused on some of the functional crosstalk that neutrophils are known to engage in with other leukocytes. Firstly, Bernson et al. extended our knowledge on the molecular basis underlying the capacity of natural killer (NK) cells to trigger neutrophil apoptosis under inflammatory conditions, by performing a comprehensive characterization of neutrophil expression of ligands for NK cell receptors. Ultimately, the authors demonstrated that inflammatory neutrophils, including either blood neutrophils stimulated *in vitro* or *in vivo*-transmigrated neutrophils, become more susceptible to NK cell-mediated apoptosis than resting neutrophils. Furthermore, their enhanced sensitivity is associated with a pronounced downregulation of HLA class I expression, which is known to release activating signaling by NK cells (Bernson et al.). Secondly, Aarts et al. compared the immunosuppressive capacity of circulating neutrophils from healthy donors (HDs) with those displayed by autologous bone marrow (BM) myeloid cell fractions at various differentiation stages. They show that, unlike “early mature” BM neutrophils, immature BM neutrophils, *per se*, are not efficient in suppressing T cells when activated with physiological stimuli (Aarts et al.). The authors therefore speculate that, for instance in cancer patients, functional granulocyte-myeloid-derived suppressor cells (g-MDSCs) require neutrophil differentiation from immature populations in the bloodstream, which are unable to perform any immunosuppressive activity in their own right (Aarts et al.). In another article, Oberg et al., first, exhaustively review current knowledge on the reciprocal interactions between neutrophils, tumor cells, and particularly gamma/delta T cells. They then provide data on the modulation of anti-tumor cytotoxicity of short-term expanded human gamma/delta T cell lines by autologous freshly isolated neutrophils. Their results demonstrate that, under certain circumstances, the presence of neutrophils can enhance, rather than inhibit, the killing capacity of gamma/delta T cells, by increasing their release of cytotoxic mediators (Oberg et al.). Finally, the role of neutrophils in malignancy was extensively reviewed by Lecot et al., highlighting the heterogeneity of neutrophils, and their effector functions contributing to cancer suppression as well as progression. The authors also reviewed different strategies aimed at targeting neutrophils in malignancy, ranging from neutrophil depletion to succinct targeting of recruitment or other key effector functions.

In conclusion, neutrophils are heterogeneous cells with a huge range of effector functions partaking in shaping our immune system. The current papers included in this Research Topic highlights the emerging role of neutrophils as communicators in health and disease with promises of future therapies targeting these pathways.

## Author Contributions

All authors listed have made a substantial, direct and intellectual contribution to the work, and approved it for publication.

## Conflict of Interest

The authors declare that the research was conducted in the absence of any commercial or financial relationships that could be construed as a potential conflict of interest.
